# Breastfeeding knowledge, attitude, and self-efficacy among mothers with infant and young child in rural Ethiopia

**DOI:** 10.1371/journal.pone.0279941

**Published:** 2022-12-30

**Authors:** Abraham Tamirat Gizaw, Pradeep Sopory, Sudhakar Morankar

**Affiliations:** 1 Department of Health, Behavior, and Society, Faculty of Public Health, Institute of Health, Jimma University, Jimma, Ethiopia; 2 Department of Communication, Wayne State University, Detroit, MI, United States of America; KMCH Institute of Health Sciences and Research, INDIA

## Abstract

**Background:**

Breastfeeding has several benefits for both mothers and their children. Despite strong evidence in support of the practice, its prevalence has remained low worldwide, particularly in Ethiopia. Therefore, this study is aimed to assess breastfeeding knowledge, attitude, and self-efficacy among mothers with index infants and young children in the rural community of Southwest Ethiopia.

**Methods:**

A community-based cross-sectional study was conducted between March and April 2022 as baseline data for a cluster of randomized control trials. Multistage sample techniques followed by systematic random sampling techniques were employed. The Chi-square and Fisher’s exact probability tests were used to assess the baseline differences in the socio-demographic characteristics of the two groups. An independent sample t-test was used to determine the mean differences. Multivariate logistic regression analysis was used to evaluate the association. All tests were two-tailed, and a statistically significant association was declared at a *p*-value ≤ 0.05.

**Results:**

A total of 516 mothers (258 from the intervention and 258 from the control group) were interviewed. A total of 516 mothers (258 from the intervention group and 258 from the control group) were interviewed. Except for the child’s sex and age, no significant difference was observed between the intervention and control groups in terms of socio-demographic variables (p > 0.05). Independent t-tests found no significant difference between the two groups (p > 0.05) in terms of the mean score of maternal breastfeeding knowledge, attitude and self-efficacy at baseline. After adjusting for other covariates, maternal age (AOR = 1.44, 95% CI: 0.69, 3.07), educational status (AOR = 1.87, 95% CI: 0.56,2.33), occupation (AOR = 1.79, 95% CI, 1.04, 3.69), ANC (antenatal care) (AOR = 1.88, 95% CI, 1.11, 4.09), received breastfeeding information (AOR = 1.69, 95% CI, 1.33, 5.04), postnatal care (PNC) (AOR = 3.85, 95% CI, 2.01, 5.77) and parity (AOR = 2.49, 95% CI, 1.08, 4.19) were significantly associated high level breastfeeding knowledge. The positive attitude was associated with maternal age (AOR = 2.41, 95% CI, 1.18, 5.67), education status (AOR = 1.79, 95% CI, 0.99,4.03), ANC (AOR = 2.07, 95% CI, 1.44,5.13), last child breastfeeding history (AOR = 1.77, 95% CI, 1.21,4.88) and high level of breastfeeding knowledge (AOR = 2.02, 95% CI, 1.56,4.04). Finally, high breastfeeding self-efficacy was associated with ANC (AOR = 1.88, 95% CI 1.04,3.83), parity (AOR = 4.05, 95% CI, 1.49, 5.03) and high knowledge level (AOR = 1.69, 95% CI, 0.89,2.85).

**Conclusions:**

The study concluded that mothers in both the intervention and control groups have a low level of breastfeeding knowledge, a neutral attitude, and medium self-efficacy. Therefore, nutrition education interventions using tailored messages appropriate to the sociocultural context in the rural setting should be developed and evaluated continuously.

## Introduction

Globally, the practice of breastfeeding is declining even though the WHO recommends, infants should be exclusively breastfed for the first 6 months and continue it to the age of 24 months to achieve optimal growth, development, and health [1]. Inappropriate breastfeeding, especially a lack of exclusive breastfeeding during the first half-year of life, is an important risk factor for infant and childhood morbidity and mortality [2, 3]. There is strong evidence that human milk feeding decreases the incidence and severity of a wide range of infectious diseases [4]. Maternal/caregiver breastfeeding knowledge, attitude, and self-efficacy are the main influencers on a child’s nutritional status [5–7].

Studies show knowledge, attitude, self-efficacy, cultural practices, maternal age, educational status, and literacy are among the most prominent factors affecting breastfeeding [8, 9]. Sub-Saharan Africa has one of the lowest prevalences of breastfeeding, which accounts for 37% of infants aged less than six months being exclusively breastfed, and diarrhea remains a significant source of morbidity and mortality among children under-5 years [10]. Plausible reasons attributable to suboptimal breastfeeding practice in Africa included lower socioeconomic status, home birthing, culture, and poor implementation and monitoring [11, 12].

In Ethiopia, breastfeeding is below the recommended infant and young child feeding practices. Current interventions such as the sustainable development goals (SDG 3 and 4), advocating for improved nutrition and healthy lives for all, and Global Nutrition Target by 2025 are initiatives needed on a large scale to reduce diarrhea mortality in resource constraint settings [10]. The practice of prelacteal feeding, likewise, decreased from 29% in 2005 to 27% in 2011 and dropped further to 8% in 2016 [13]. A study conducted in the Dabat Health and Demographic Surveillance System site; in northwest Ethiopia also showed that the prevalence of early initiation of breastfeeding was 43.9%. The most common prelacteal food given was raw butter (49.1%) [14]. The study conducted in southwest rural Ethiopia showed that redrafted reasons for terminating breastfeeding were pregnancy (33.9%), not having enough breast milk (16.1%), and being tired of breastfeeding (12.6%) [5].

With the intent of improving breastfeeding rates in rural Ethiopia by raising the cognitive and affective domains of mothers towards breastfeeding. We conducted this study as a baseline to measure levels and identify predictors of knowledge, attitude, and breastfeeding self-efficacy among mothers. This study contributes new insight into the factors that influence knowledge, attitude, and breastfeeding self-efficacy, using validated instruments.

## Methods

### Study setting and period

This study was conducted in Maji Woreda, one of the rural settings of the West Omo Zone, Southwest Ethiopia. Maji Woreda has a total of 22 kebeles. The woreda has one district hospital, two health centres, and 22 health posts. Maji district has a population of 230,777 people and is located 817 kilometres away from Addis Ababa, Ethiopia [15].

### Study design and population

This is a community-based cross-sectional study that used data collected from March 1 to April 3, 2022, as part of a cluster-randomized control trial baseline. The study populations were mothers with infants and children aged 0–24 months in randomly selected small administrative units.

### Sample size determination

This study is a part of a larger study entitled " Effectiveness of a positive deviance approach to improve appropriate feeding and nutritional outcomes in South West Region, Ethiopia: a cluster randomized controlled trial".

WHO Trial Registration number: PACTR202108880303760.

The sample size was calculated using statcalc with the following assumptions: to detect an increase in appropriate feeding from 7% to 14% [16], with 95% CIs and 80% power, assuming an intra-class correlation coefficient of 0.03 [17] equal to the Ethiopian study for a cluster size of 12, it was calculated that 36 clusters were needed. This gave a sample size of 215. Then it was multiplied by the design effect of two and allowing for a 20% loss to follow-up, the total sample size was 516 mothers (258 from the intervention arm and 258 from the control arm).

### Sampling and randomization

A multistage sampling technique followed by a systematic random sampling technique was used to identify mothers with index infants and young children. In the first step, one woreda (district) was selected by simple random sampling (lottery method). Second, lists of all kebeles (clusters) in the selected districts were compiled from the district administrative offices. A total of 36 non-adjacent clusters geographically accessible out of the 88 zones (small administrative units) were purposefully selected by listing them in alphabetical order then a list of random numbers was generated in Microsoft Excel 2016 and fixed by being copied as "value" next to the alphabetical list of zones. According to the produced, random numbers were placed in ascending order. The last 18 zones were chosen as control clusters, and the first 18 served as intervention clusters. Third, 516 mothers were recruited using health extension workers’ family registration books to find mothers who had an infant and a young child with an age less than 24 months. An Excel sheet was formed from the logbook, and the households were selected using simple random sampling techniques.

Mothers within the zones serve as the unit of observation, and zones in the kebeles serve as the unit of randomization for the trials. The zones are assigned by simple randomization with a 1:1 allocation to either the control or intervention groups. The intervention assignment was concealed from the interviewers collecting the outcome data. Because of the nature of the intervention, mothers cannot be blind. All mothers, health extension workers, members of the women’s health army, and community volunteers, however, are blind to the study’s hypothesis. The general objectives of the study are described in the agreement for data collection.

### Inclusion and exclusion criteria

#### Inclusion criteria

Mothers living in the selected clusters with no plan to move away during the intervention period, capable of giving informed consent, willing to be visited by supervisors and data collectors, the mothers should have an infant/child aged 0–24 months, the child Height-for-age Z (HAZ) scores HAZ < −2 and child with no severe malnutrition.

### Exclusion criteria

Mothers with a severe psychological illness and children with severe illness were excluded.

### Data collection

Data were collected on socio-demographics, knowledge, attitude, breastfeeding self-efficacy, and household food security status from all participants. The questionnaire was prepared in English, translated to the local language Amharic and then back-translated to English by experts in the language to maintain its consistency. The data were collected using an interviewer-administered questionnaire in Amharic. Three BSc nurses and ten diploma holders in the health centre were recruited as supervisors and data collectors, respectively. The supervisors were supervised and coordinated with their respective kebeles. Pretesting of the questionnaire was done on 5% of mothers who had an infant and child aged 0–24 months in another area (the Bench-Sheko zone) before the study period, and appropriate changes were made to the questionnaire.

### Measurements

A questionnaire was developed from a large body of literature to address the survey objectives. This instrument was tested for reliability and validity (Cronbach’s alpha coefficients were 0.84, 0.87, and 0.77 for breastfeeding knowledge, attitude, and self-efficacy, respectively). Household food security tools were also used to assess the food security status of the household. A self-report questionnaire was used, which consisted of four sections: socio-demographic characteristics (maternal age, marital status, educational level, and monthly income), perinatal characteristics (delivery mode, birth order, and weight of infant/young child), and breastfeeding-related characteristics (previous breastfeeding experience) [18].

We used a breastfeeding knowledge questionnaire (BFKQ) consisting of 17 items to measure the knowledge of the participants about breastfeeding. There are three possible responses for each item (true, false, and I do not know or not sure). Correct responses were scored as one, and zero for other options. Thus, the total scores ranged from 0–17, these items were developed based on a study done among Chinese mothers in English and translated into Amharic [19]. We decided to use cut-offs above and below the mean to dichotomize knowledge level. Accordingly, all mothers who scored ≥ the mean in the knowledge test were considered to have a high level of knowledge, and those scoring below the mean were considered to have a low level of knowledge.

The Iowa infant feeding attitude scale, Amharic version (IIFAS-A), consists of 17 items with a five-point Likert scale, rating maternal attitude towards breastfeeding. A sum of scores ranging from 17 to 85, with the higher score reflecting a positive attitude and the opposite score showing a negative attitude. Attitude toward breastfeeding was categorized as follows: (1) positive to breastfeeding (IIFAS score 70–85), (2) neutral (IIFAS score 49–69), and (3) positive to formula feeding (IIFAS score 17–48). The scale consists of 17 items with a five-point Likert scale ranging from strongly disagree to strongly agree on each item to indicate attitude toward infant feeding. The IIFAS is a validated and reliable measure (Cronbach’s alpha scores range from (0.81–0.86)) that evaluates breastfeeding attitudes in different cross-cultural settings [19–27]. Approximately half of the questions were negatively worded (i.e., 1, 2, 4, 6, 8, 10, 11, 14, and 17) [20].

The short form of the breastfeeding self-efficacy scale (BSES-SF) has been used widely with a variety of populations and published in different reputable journals [21, 22]. The overall score of the scale was calculated as the mean score of all items. A higher total score is indicative of a greater level of maternal breastfeeding self-efficacy. We used the BSES-SF, consisting of 14 items with a five-point Likert scale, developed to measure breastfeeding confidence in Amharic translated from validated English questionnaires from different studies, which measures the mother’s self-efficacy in her ability to breastfeed. All the items are preceded by the phrase “I can always “and anchored with a 5-point Likert scale where one indicates not at all confident and five indicates always confident. All items are presented positively, and scores are summed to produce a range from 14 to 70. Breastfeeding self-efficacy is identified from the sum of each question: low self-efficacy (14 to 32 points), medium self-efficacy (33 to 51 points), and high self-efficacy (52 to 70 points) [23].

The Household food insecurity access scale (HFIA) was assessed by using 9-item questions. Before assigning the food insecurity category, each frequency of occurrence response was coded as 0 for all cases where the answer to the corresponding occurrence question was "no," and the four food security categories were computed and created in the order recommended by FANTA [24]. Finally, HFIA category 1 was considered food secure, while the remaining categories were considered food insecure.

### Data analysis

Double data entries were done using EpiData (version 3.1), and all statistical analyses were conducted using SPSS version 23. The data were summarized using frequencies and percentages. The Chi-square and Fisher’s exact probability tests were used to assess the baseline differences in the socio-demographic characteristics of the two groups. An independent sample t-test was used to determine the mean differences in breastfeeding knowledge, attitude, and self-efficacy. Logistic regression analysis was conducted to assess the association between the outcome variables and the covariates. Variables in the univariate analysis that showed a significant effect on the dependent variable were included in the multivariable analysis. Unadjusted and Adjusted Odds ratios (AOR) with 95% confidence interval (95% CI) were computed. All tests were two-tailed, and a statistically significant association was declared at a *p*-value ≤ 0.05.

### Ethical approval and consent to participate

The study received ethical approval (reference number. IHRPG/938/20) from Jimma University’s Institute of Health Research and Postgraduate Office, Institutional Review Board. Administrative permission was acquired from Maji Woreda Administrative offices, and formal letters to the research area were obtained from Maji Woreda Health Office. All participants provided written informed consent. Participation was voluntary, with the right to withdraw at any time.

## Results

### Baseline socio-demographic characteristics of participants

In this study, 516 mothers (258 from the intervention and 258 from the controls) were interviewed providing a 100% of overall response rate. The mother’s mean (±SD) age was 31.69 (±7.74) for the intervention and 30.83 (±7.01) for the control groups respectively. The majority of the mothers were married (96.5%), housewives (99.0%), with primary school education (55.62%), and lived in a food-insecure household (81.2%). Two hundred thirty-four (45.35%) women had no antenatal care (ANC) visits, and only 11.05% had the recommended *≥* 4 ANC visits. Regarding child-related variables, the mean age was 10.97 (± 4.96) months for the intervention and 11.39(± 5.80) for the control groups. As shown in Table 1, except for child sex and child age, there were no statistically significant differences between the intervention and the control groups at baseline.

**Table 1:** Socio-demographic characteristics of mothers, in Southwest Ethiopia.

Variables	Intervention (n = 258)		Control (n = 258)		X^2^ test	p-value
	n	(%)	n	(%)		
**Mother’s age (in years)**						
18–24	38	14.23	38	14.23	0.07	0.967
25–34	130	51.55	136	51.55		
35–49	90	34.22	84	34.22		
M±SD	31.69 ± 7.74		30.83 ± 7.01			
**Marital status**						
Married	247	95.74	251	97.30	0.92	0.337
Divorced	11	4.26	7	2.70		
**Religion**						
Orthodox Christian	166	64.34	171	66.28	0.21	0.644
Protestant	92	35.66	87	33.72		
**Maternal occupation** ^**†**^						
Housewife/farmer	256	99.22	255	98.84		0.999
Government employee	2	0.78	3	1.16		
**Family size**						
1–3	58	22.48	58	22.48	3.02	0.221
4–6	131	50.77	147	56.98		
≥7	69	26.75	53	20.54		
**Monthly income of the household (ETB)**						
≤500	140	54.26	124	48.06	2.53	0.639
500–1000	90	34.88	98	37.98		
1000–1500	14	5.43	16	6.20		
1501–2000	8	3.10	12	4.65		
≥2000	6	2.33	8	3.11		
**Maternal educational status**						
Illiterate	97	37.58	91	35.27	0.30.	0.859
Primary school	141	54.65	146	56.59		
Secondary school and higher	20	7.77	21	8.14		
**Household food security status**						
Secured	14	5.43	13	5.04	0.04	0.843
Not secured	244	94.57	245	94.96		
**How long did you breastfeed your last child**						
<24 months	152	58.91	165	63.95	1.38	0.24
≥24 months	106	41.09	93	36.05		
**Child sex**						
Male	157	60.85	131	50.77	5.31	0.021*
Female	101	39.15	127	49.23		
**Child age (months)**						
0–5	41	15.89	45	17.44	15.03	0.002*
6–11	85	32.94	85	32.94		
12–17	118	45.74	90	34.88		
18–24	14	5.43	38	14.74		
M±SD	10.97 ± 4.96		11.39 ± 5.80			
**Birth order**						
1^st^	45	17.44	44	17.05	0.09	0.958
2^nd^ - 4^th^	171	66.28	174	67.44		
5^th^ or more	42	16.28	40	15.51		
**Number of ANC visits**						
No ANC visits	118	45.74	116	44.96	0.04	0.981
<4 visits	112	43.41	113	43.8		
≥4 visits	28	10.85	29	11.24		
**Received breastfeeding information**						
No	174	67.44	179	69.38	0.22	0.636
Yes	84	32.56	79	30.62		
**Place of delivery**						
Home	173	67.05	184	71.32	1.1	0.294
Health institution	85	32.95	74	28.68		
**Delivery type** ^**†**^						
Normal vaginal delivery	254	98.45	255	98.84		0.999
Caesarian section	4	1.55	3	1.16		
**First time breastfeeding**						
Immediately/within an hour of birth	91	35.27	98	37.98	0.87	0.833
After the first hour	130	50.39	127	49.22		
After one day	11	4.26	12	4.65		
Do not remember/ do not know	26	10.08	21	8.15		
**Postpartum complications**						
No	185	71.70	193	74.81	0.63	0.426
Yes	73	28.30	65	25.19		
**Postnatal care**						
No	214	82.94	225	87.21	1.85	0.174
Yes	44	17.06	33	12.79		
**Number of children**						
1–2	50	19.38	58	22.48	2.11	0.349
3–4	130	50.39	136	52.71		
≥5	78	30.23	64	24.81		
**Parity**						
Primiparous	45	17.44	44	17.05	0.01	0.907
Multiparous	213	82.56	214	82.95		

Chi^2^ test

*significant at p < 0.05

^†^Fisher’s exact probability test

### Baseline breastfeeding knowledge, attitude and self-efficacy among the groups

Regarding the outcome variables, there were no statistically significant differences between the intervention and control groups were observed at the baseline (Table 2).

**Table 2:** Baseline comparison of the mean total breastfeeding knowledge, attitude and self-efficacy scores of the intervention and control groups.

Breastfeeding variables	Mean ± SD		t-test	Mean difference(95%CI)	P-value
	Intervention (n = 258)	Control (n = 258)			
Knowledge	6.67 ± 2.03	6.76 ± 2.04	-0.07	-0.09 (-2.94, 2.74)	0.913
Attitude	54.88± 3.38	52.53± 3.07	0.375	2.35 (1.84,2.67)	0.754
Self-efficacy	34.39 ±5.20	34.54 ± 5.36	-0.06	-0.15 (-1.52,1.42)	0.997

### Predictors of breastfeeding knowledge

In an unadjusted model, age, educational status, occupation, number of ANC visits, breastfeeding information received, postnatal care, parity, attitude, and self-efficacy were significantly associated with a high level of breastfeeding knowledge. After adjusting for other covariates, mothers in the age group of 24–35 were more likely to have a high level of knowledge (AOR = 1.44, 95% CI: 0.69, 3.07) compared to those who were in the age group of 18–24. Compared to illiterate mothers, those who completed secondary school and higher were more likely to have a high level of knowledge (AOR = 1.87, 95% CI: 0.56,2.33). Also compared with mothers who were housewives/farmers, mothers who were government employees were more likely to have a high level of knowledge (AOR = 1.79, 95% CI, 1.04, 3.69). Similarly compared to mothers who had no ANC visits, mothers who had ≥ 4 visits were more likely to have a high level of knowledge (AOR = 1.88, 95% CI, 1.11, 4.09). Those who received breastfeeding information and postnatal care were more likely to have a high level of knowledge (AOR = 1.69, 95% CI, 1.33, 5.04) and (AOR = 3.85, 95% CI, 2.01, 5.77) compared with those who did not receive information and no postnatal visits, respectively. Multiparous mothers were more likely to have a high level of knowledge compared with primiparous mothers (AOR = 2.49, 95% CI, 1.08, 4.19) (Table 3).

**Table 3:** Association between breastfeeding knowledge and their correlates.

Variables	n (%)	Unadjusted OR (95% CI)	Adjusted OR (95% CI)
**Mother’s age (in years)**			
18–24	76 (14.7)	1	1
25–34	266 (51.5)	1.11 (0.87, 2.62)*	1.44 (0.69,3.07) *
35–49	174 (33.8)	1.22 (1.01,3.09)	1.55 (1.33,5.53)
**Maternal educational status**			
Illiterate	188 (36.4)	1	1
Primary school	287 (55.7)	3.21 (1.41,6.99)	3.24 (1.29,4.66)
Secondary school and higher	41 (7.9)	1.78 (1.22,6.11) *	1.87 (0.56,2.33)*
**Maternal occupation**			
Housewife/farmer	511 (99.0)	1	1
Government employee	5 (1.0)	1.67 (1.03,2.98)	1.79 (1.04,3.69)*
**Number of ANC visits**			
No ANC visits	234 (45.3)	1	1
<4 visits	225 (43.6)	2.12 (1.19,5.16)*	2.21 (1.82,4.77)
≥4 visits	57 (11.1)	1.44 (0.99, 4.03) *	1.88 (1.11,4.09) **
**Received breastfeeding information**			
No	353 (68.4)	1	1
Yes	163 (31.6)	1.63 (1.08,3.69)*	1.69 (1.33,5.04) *
**Postnatal care**			
No	439 (85.1)	1	1
Yes	77 (14.9)	2.52 (1.25,3.69)*	3.85 (2.01,5.77) *
**Parity**			
Primiparous	89 (17.2)	1	1
Multiparous	427 (82.8)	2.36 (1.11,4.57)*	2.49 (1.08,4.19)**
**Breastfeeding attitude**			
Negative	182 (35.27)	1	1
Neutral	239 (46.32)	1.23 (0.98,3.98)	1.34 (1.17,3.25)
Positive	95 (18.41)	2.18 (1.98,3.55)*	2.15 (1.54,4.01)
**Breastfeeding self-efficacy**			
Low	96 (18.60)	1	1
Medium	242 (46.90)	2.87 (1.99,5.78)	2.62 (1.52,4.77)
High	178 (34.50)	3.01 (2.01, 4.33)*	2.99 (1.87,3.69)

*p<0.05

**p<0.001; ANC: Antenatal care; PNC: Postnatal care

### Predictors of breastfeeding attitude

The variables included in the multiple logistic regression model, i.e., age, educational status, number of ANC visits, last child breastfeeding history, parity, and high-level knowledge were significantly associated with a positive attitude (Table 4). Mothers in the age range of 25–34, as compared to their younger counterparts (18–24) were more likely to have a positive attitude towards breastfeeding (AOR = 2.41, 95% CI, 1.18, 5.67). Similarly, those with secondary school and higher education (AOR = 1.79, 95% CI, 0.99,4.03) compared with illiterate, ≥ 4 ANC visits (AOR = 2.07, 95% CI, 1.44,5.13) compared with no ANC visits, mothers who breastfed their child ≥24 (AOR = 1.77, 95% CI, 1.21,4.88) months compared with mothers who breastfed ≤24 months and mothers with a high level of breastfeeding knowledge (AOR = 2.02, 95% CI, 1.56,4.04) compared with their counterparts were more likely had a positive attitude toward breastfeeding.

**Table 4:** Association between breastfeeding attitudes and their correlates.

Variables	n (%)	Unadjusted OR (95% CI)	Adjusted OR (95% CI)
**Mother’s age (in years)**			
18–24	76 (14.7)	1	1
25–34	266 (51.5)	2.25 (1.22,4.08)*	2.41 (1.18,5.67)*
35–49	174 (33.8)	1.28 (1.01,3.19)	1.35 (1.02,3.68)
**Maternal educational status**			
Illiterate	188 (36.4)	1	1
Primary school	287 (55.7)	1.02 (0.81,2.94)	1.71 (1.11,2.94)
Secondary school and higher	41 (7.9)	1.68 (0.92, 2.95)*	1.79 (0.99,4.03)*
**Maternal occupation**			
Housewife/farmer	511 (99.0)	1	1
Government employee	5 (1.0)	1.19 (0.88, 2.99)*	1.66 (1.01,4.02)
**Number of ANC visits**			
No ANC visits	234 (45.3)	1	1
<4 visits	225 (43.6)	1.03 (0.79,4.36)	1.22 (0.89,3.13)
≥4 visits	57 (11.1)	1.73 (1.13,3.81)**	2.07 (1.44,5.13)**
**Last child’s breastfeeding history(months)**			
<24	353 (68.4)	1	1
≥24	163 (31.6)	1.45 (1.01,4.57) *	1.77 (1.21,4.88) *
**Postnatal care**			
No	439 (85.1)	1	1
Yes	77 (14.9)	2.52 (1.61,3.01)*	2.88 (1.21,5.44)**
**Parity**			
Primiparous	89 (17.2)	1	1
Multiparous	427 (82.8)	1.18 (0.62,2.02) *	1.52 (1.06,3.06) *
**Breastfeeding knowledge**			
Low	277 (53.69)	1	1
High	239(46.31)	189 (1.22, 3.85) *	2.02 (1.56,4.04) **
**Breastfeeding self-efficacy**			
Low	96 (18.60)	1	1
Medium	242 (46.90)	1.08 (0.85,3.01)	1.55 (0.97,3.69)
High	178 (34.50)	2.17 (1.01,4.32)*	2.28 (0.94,3.71)

*p<0.05

**p<0.001

### Predictors of breastfeeding self-efficacy

In an unadjusted model, age, educational status, number of ANC visits, parity, breastfeeding knowledge and attitude were significantly associated with high breastfeeding self-efficacy. After adjusting for other covariates, mothers who attended ≥4 ANC visits were more likely had higher self-efficacy (AOR = 1.88, 95% CI 1.04,3.83) compared with mothers with no ANC visits. Multiparous mothers were more likely had higher self-efficacy (AOR = 4.05, 95% CI, 1.49, 5.03) compared with the primiparous. Similarly, mothers with high knowledge level (AOR = 1.69, 95% CI, 0.89,2.85) were high self-efficacy compared with their counterparts (Table 5).

**Table 5:** Association between breastfeeding self-efficacy and their correlates.

Variables	n (%)	Unadjusted OR (95% CI)	Adjusted OR (95% CI)
**Mother’s age (in years)**			
18–24	76 (14.7)	1	1
25–34	266 (51.5)	1.82 (1.01,2.99) *	1.91 (1.22,3.07)
35–49	174 (33.8)	0.73 (0.61, 1.86)	0.69 (0.33,1.45)
**Maternal educational status**			
Illiterate	188 (36.4)	1	1
Primary school	287 (55.7)	1.44 (1.03,2.55)	1.59 (1.23,3.11)
Secondary school and higher	41 (7.9)	1.32 (0.89, 2.09)*	1.98 (1.08, 3.85)
**Number of ANC visits**			
No ANC visits	234 (45.3)	1	1
<4 visits	225 (43.6)	1.19 (0.79, 1.74)	1.34 (1.01, 2.22)
≥4 visits	57 (11.1)	1.39 (1.03,3.01)*	1.88 (1.04,3.83) *
**Parity**			
Primiparous	89 (17.2)	1	1
Multiparous	427 (82.8)	3.31 (1.91, 5.02)*	4.05 (1.49, 5.03)*
**Breastfeeding knowledge**			
Low	277 (53.69)	1	1
High	239 (46.31)	1.29 (0.83, 2.63) *	1.69 (0.89,2.85)**
**Breastfeeding attitude**			
Negative	182 (35.27)	1	1
Neutral	239 (46.320	1.09 (0.79,2.84)*	1.19 (0.89, 2.98)
Positive	95 (18.41)	2.08 (1.55, 2.56)*	2.44 (1.19,3.27)

*p<0.05

**p<0.001

## Discussion

Breastfeeding practice by mothers is influenced by many factors, such as breastfeeding knowledge, attitude, and self-efficacy, as well as socio-demographic factors (e.g., age of the mother, educational status, occupation, antenatal visits, information obtained, and past experiences), Even though Ethiopia has attempted to increase breastfeeding for children 0–2 years, its success has been limited. To the best of our knowledge, there are only a few studies that have used validated questionnaires to assess breastfeeding knowledge, attitude, and self-efficacy in Ethiopia and particularly in the study area. As a result, the baseline survey provides a novel insight into the study of breastfeeding self-efficacy among mothers, which has not previously studied in the region.

Notably, significant proportions of mothers in the study area had a low level of breastfeeding knowledge. In this setting improving awareness among mothers could be a major step towards optimal breastfeeding. In Ethiopia, this may be imperative to help reach the target set in the national health sector plan [24]. This baseline study revealed that the mean knowledge score was (6.67 ± 2.03) for the intervention and (6.76 ± 2.04) for the control respectively, much lower than the study done among mothers in Oromia, Ethiopia [25], and China [19]. This difference may be due to factors such as a lack of adequate information received and awareness regarding the importance of breastfeeding. Further, the nature of our study setting is remote as compared to the above studies and majority of mothers has limited exposure to medias.

In this study, mothers in the age group of 25–34, having completed secondary school or higher, being government employees, having 4 ANC follow-ups, receiving breastfeeding information, having postnatal care, and being multiparous showed a significant association with a high level of knowledge. This mirrors the findings in Ethiopia [27, 28], developing [1, 29–34], as well as developed countries [35–38]. The majority of the mothers in the age group 25–34 have the educational status of secondary school or higher, government employee, those mothers in the ANC follow-up, received breastfeeding information, had postnatal care and multiparous had exposure to breastfeeding information mainly from the health professional; these could probably be reasons for a high level of breastfeeding knowledge as compared to their counterparts.

This study revealed that the mean IIFAS scores were (54.88 ± 3.38) for the intervention and (52.53 ± 3.07) for the control, which is within a range reflecting a neutral attitude towards breastfeeding. The findings of the current study are in congruence with similar findings from Ethiopia [20] and other countries, including China [19], Italy [39], and India [7, 40]. However, this study is contrary to the study conducted in Australia, which had a positive IIFAS score [41]. This difference may result from exposure to information from different sources, and health literacy status could probably contribute to this discrepancy. Also, the findings of this study are somewhat promising compared to the study conducted in Northwest Ethiopia [42], in which mothers had a negative attitude.

In addition, this study revealed that a positive breastfeeding attitude was associated with maternal age (25–34), educational status (secondary education and higher), occupation (government employee), ≥4 ANC visits, last child breastfeeding experience (≥24 months), postnatal care, parity (multiparous), and high level of breastfeeding knowledge. The findings from the current study is in congruence with similar findings in the literature, such as maternal age, educational status, and occupation, which have been reported in Ethiopia [26], China [19], and Spain [43]. The mothers in the age group of 25–34 had a positive attitude as compared to those mothers younger than them. This may be due to mothers in the age group of 25–34 having exposure to the benefits of breastfeeding from health professionals. The literature that had similar findings for ≥4 ANC visits, last child breastfeeding experience (≥24 months), postnatal care, and parity (multiparous) as predictors of a positive breastfeeding attitude has been reported in different studies in Ethiopia [33, 34] and Spain [43]. Finally, high levels of breastfeeding knowledge were associated with a positive attitude; this study is in line with the studies done in Ethiopia [26, 27] and Poland [44].

A mother’s confidence is also a strong indicator of breastfeeding practices, according to different studies conducted across the globe [45, 46]. The current study showed that the mean BSES-SF score was (34.39±3.38) for the intervention and (34.54±5.36) for the control group, which was lower than the study done in Greece [45], Turkey [47], Saudi Arabia [48], and Jordan [49]. The discrepancy in the result may be due to the limited media exposure in the study area, and the lack of visual demonstration of breastfeeding through electronic media could probably be the reason for low self-efficacy. The current study showed that there was a significant association between high breastfeeding self-efficacy with number of ANC visits (≥ 4 ANC visits), parity (primiparous) and a high level of breastfeeding knowledge.

In this study, high breastfeeding self-efficacy was associated with the number of ANC visits (≥4 visits). This study was in line with the studies conducted in Brazil [23] and Nepal [50]. The possible explanation for the association between high breastfeeding self-efficacy and ANC visits may be due to increased exposure of mothers to health education from the health professional during their visit to the health care.

This study revealed that high breastfeeding self-efficacy was significantly associated with parity (multiparous). This may be due to their experiences and abilities to solve breastfeeding problems, which could possibly contribute to high breastfeeding self-efficacy. The finding of this study is in line with a study conducted in Denmark [51]. High breastfeeding knowledge has been identified as a predictor of breastfeeding self-efficacy. A possible explanation could be that mothers received information on the benefits of breastfeeding and exposure to health information during their visit to the ANC follow-up clinic, and knowledge obtained in a social environment boosted their self-efficacy. Our finding is also congruence with previous studies from Brazil [23], Spain [43], and Nepal [50].

### Strength and limitations of the study

The study’s strength includes the use of pretested and validated tools for the study. The implementation of day-to-day supervision during data collection periods was study strength. This study has the following limitations. First, the survey is a cross-sectional design; thus, causal relationships cannot be established. Second, the study was conducted in a rural area and thus, additional research is needed to assess breastfeeding knowledge, attitude, and self-efficacy and its predictors in urban settings. Third, a qualitative study should be conducted to explore barriers and facilitators to breastfeeding.

## Conclusion

This study showed that a significant proportion of rural mothers have a low level of breastfeeding knowledge, a neutral attitude, and medium self-efficacy. Based on the results of this study, the following suggestions are provided: further research, education, and policies for promoting breastfeeding in rural and remote settings. Furthermore, nutrition education interventions using tailored messages and appropriate to the sociocultural context in the rural setting should be developed and evaluated continuously.

## Supporting information

S1 Table Descriptive statistics of breastfeeding knowledge, attitude and self-efficacy. (DOCX)

S1 Data (XLS)
