# Supplementary material for: Breastfeeding knowledge, attitude, and self-efficacy among mothers with infant and young child in rural Ethiopia
Source: PLoS One. 2022 Dec 30;17(12):e0279941. doi: 10.1371/journal.pone.0279941 (PMC9803198; doi:10.1371/journal.pone.0279941)
Supplement: S1 Table — (DOCX) [file pone.0279941.s001.docx]

**Descriptive statistics of breastfeeding Knowledge among the intervention and control groups**

| Items (correct response) | Intervention (n= 258) | | Control (n=258) | |
| --- | --- | --- | --- | --- |
|  | **n** | **(%)** | **n** | **(%)** |
| Breastfeeding can reduce the incidence of maternal ovarian cancer and breast disease (true) | 35 | 13.56 | 33 | 12.79 |
| Breast milk enhances infant immunity to infections and childhood diseases like asthma (true) | 60 | 23.25 | 62 | 24.03 |
| The risk of obesity is relativity low for breastfed children (true) | 51 | 19.77 | 51 | 19.77 |
| Recommended duration of exclusive breastfeeding is six months (true) | 209 | 81.01 | 207 | 80.23 |
| A reliable sign an infant is fed adequately is based on the daily urine amount (true) | 100 | 38.76 | 109 | 42.25 |
| After breastfeeding, milk should not be left in the breast (false) | 47 | 18.22 | 36 | 13.95 |
| Breastfeeding is useful for spacing between children’s births (true) | 58 | 22.48 | 58 | 22.48 |
| Baby needs to be breastfed on a schedule (false) | 15 | 5.81 | 12 | 4.65 |
| Breastfeeding can provide adequate nutrition for a 6 month-old infant without other food or beverages (true) | 208 | 80.62 | 203 | 78.68 |
| Infant formula is as good as or even better than human milk (false) | 201 | 77.91 | 202 | 78.29 |
| Breastfeeding can reduce the incidence of postpartum hemorrhage (true) | 62 | 24.03 | 60 | 23.25 |
| Breastfeeding can promote postpartum uterine contraction, weight loss, and delay return of menses (true) | 95 | 36.82 | 90 | 34.88 |
| The more the baby sucks, the mother’s milk production is greater (true) | 208 | 80.62 | 211 | 81.78 |
| Breastfeeding can promote an infant’s intellectual development and growth (true) | 155 | 60.08 | 144 | 55.81 |
| Breastfeeding can prevent infant diarrhea (true) | 133 | 51.55 | 133 | 51.55 |
| Three months of breastfeeding is long enough (false) | 178 | 68.99 | 177 | 68.60 |
| It is recommended that women start breastfeeding as soon as possible after childbirth (true) | 135 | 52.32 | 142 | 55.04 |
| n-frequency of correct responses per items | | | | |

**Descriptive statistics of breastfeeding attitude among the intervention and control groups**

| Variables | Intervention (n= 258) | | | Control (n=258) | | |
| --- | --- | --- | --- | --- | --- | --- |
|  | **Disagree**  **n (%)** | **Neutral**  **n (%)** | **Agree**  **n (%)** | **Disagree**  **n (%)** | **Neutral**  **n (%)** | **Agree**  **n (%)** |
| The beneﬁts of breastfeeding last only as long as the baby is breast-fed^†^ | 95 (36.82) | 61 (23.64) | 102 (39.54) | 94 (36.43) | 61 (23.64) | 103 (39.92) |
| Formula feeding is more convenient than breastfeeding^†^ | 165 (63.95) | 1 (0.39) | 90 (35.66) | 153 (59.30) | 12 (4.65) | 93 (36.05) |
| Breastfeeding increases mother infant bonding | 10 (3.87) | 113 (43.79) | 135 (52.34) | 9 (3.49) | 109 (42.25) | 140 (54.26) |
| Breast milk is lacking in iron^†^ | 83 (32.17) | 80 (31.01) | 95 (36.82) | 80 (31.01) | 85 (32.94) | 93 (36.05) |
| Formula-fed babies are more likely to be overfed than breastfed babies | 80 (31.01) | 43 (16.67) | 135 (52.32) | 82 (31.78) | 46 (17.24) | 130 (50.98) |
| Formula feeding is the better choice if the mother plans to go back to work^†^ | 83 (32.17) | 10 (3.87) | 165 (63.95) | 83 (32.17) | 7 (2.71) | 168 (65.11) |
| Mothers who formula feed miss one of the great joys of motherhood | 55 (21.32) | 26 (10.08) | 177 (68.60) | 44 (17.03) | 26 (10.08) | 188 (72.89) |
| Women should not breastfeed in public places such as restaurants^†^ | 140 (54.26) | 17 (6.59) | 101 (39.15) | 137 (53.10) | 11 (7.75) | 110 (42.63) |
| Breastfed babies are healthier than formula-fed babies | 30 (11.63) | 55 (21.32) | 173 (67.05) | 58 (24.48) | 56 (21.70) | 144 (55.81) |
| Breastfed babies are more likely to be overfed than formula-fed babies^†^ | 100 (38.75) | 62 (24.03) | 96 (37.22) | 99 (38.37) | 62 (24.03) | 97 (37.60) |
| Fathers feel left out if a mother breast-feeds^†^ | 162 (62.79) | 40 (15.50) | 56 (21.70) | 162 (62.79) | 46 (17.83) | 50 (19.38) |
| Breast milk is the ideal food for babies | 7 (2.71) | 25 (9.69) | 226 (87.60) | 10 (3.87) | 30 (11.63) | 218 (84.50) |
| Breast milk is more easily digested than formula | 30 (11.63) | 110 (42.63) | 118 (45.74) | 69 (26.74) | 118 (45.74) | 71 (27.52) |
| The formula is as healthy for an infant as breast milk^†^ | 110 (42.63) | 60 (23.25) | 88 (34.12) | 142 (55.04) | 63 (24.42) | 53 (20.54) |
| Breastfeeding is more convenient than formula | 23 (8.91) | 37 (14.34) | 198 (76.75) | 23 (8.91) | 40 (15.50) | 195 (75.58) |
| Breast milk is cheaper than formula | 20 (7.75) | 50 (19.38) | 188 (72.89) | 19 (7.34) | 57 (22.12) | 182 (70.54) |
| A mother who occasionally drinks alcohol should not breastfeed her baby^†^ | 40 (15.50) | 80 (31.01) | 138 (53.49) | 47 (15.89) | 85 (32.95) | 132 (51.16) |
| n-frequency, ^†^ Reverse scored items | | | | | | |

**Descriptive statistics of breastfeeding self-efficacy among the intervention and control groups**

| Variables | Intervention (n= 258) | | | Control (n=258) | | |
| --- | --- | --- | --- | --- | --- | --- |
|  | **not at all confident**  **n (%)** | **Neutral**  **n (%)** | **confident**  **n (%)** | **not at all confident**  **n (%)** | **Neutral**  **n (%)** | **confident**  **n (%)** |
| Determine that my baby is getting enough milk. | 78 (30.23) | 102 (39.54) | 78 (30.23) | 78 (30.23) | 101 (39.15) | 79 (30.62) |
| Successfully cope with breastfeeding like I have with other challenging tasks. | 75 (29.07) | 58 (22.48) | 125 (48.45) | 74 (28.68) | 58 (22.48) | 126 (48.84) |
| Breastfeed my baby without using artificial milk as a supplement. | 29 (11.24) | 100 (38.76) | 129 (50.0) | 35 (13.56) | 61 (23.65) | 162 (62.79) |
| Ensure the baby is properly lached on for the whole feeding. | 85 (32.94) | 83 (32.17) | 90 (34.88) | 78 (30.23) | 118 (45.74) | 62 (24.03) |
| Manage breastfeeding situation to my satisfaction. | 99 (38.37) | 82 (31.78) | 77 (29.85) | 87 (33.72) | 102 (39.54) | 69 (26.74) |
| Manage to breastfeeding even if my baby is crying. | 100 (38.76) | 32 (12.40) | 124 (48.84) | 66 (25.58) | 66 (25.58) | 126 (48.84) |
| Keep wanting to breastfeed. | 22 (8.52) | 45 (17.44) | 191 (74.03) | 29 (11.24) | 32 (12.40) | 197 (76.36) |
| Confortably breastfeed with my family members present. | 69 (26.74) | 78 (30.23) | 111 (43.02) | 73 (28.29) | 77 (29.85) | 108 (41.86) |
| Be satisfied with my breastfeeding experience. | 33 (12.79) | 52 (20.160 | 173 (67.05) | 29 (11.24) | 69 (26.74) | 160 (62.01) |
| Deal with the fact that breastfeeding can be time-consuming. | 24 (9.30) | 126 (48.84) | 108 (41.86) | 37 (14.34) | 112 (43.41) | 109 (42.25) |
| Finish feeding my baby on one breast before switching to the other breast. | 57 (22.12) | 92 (35.66) | 109 (42.25) | 64 (24.81) | 53 (20.54) | 141 (54.65) |
| Continue to breastfeed my baby for every feeding. | 39 (15.12) | 92 (35.66) | 127 (49.22) | 47 (18.22) | 80 (31.01) | 131 (50.77) |
| Manage to keep up with my baby’s breastfeeding demands. | 55 (21.32) | 95 (36.82) | 108 (41.86) | 41 (15.89) | 97 (37.60) | 120 (46.51) |
| Tell when my baby is finished breastfeeding. | 44 (17.05) | 121 (46.90) | 93 (36.05) | 48 (18.60) | 133 (51.55) | 77 (29.85) |
| n-frequency | | | | | | |
